# Exosites in Hypervariable Loops of ADAMTS Spacer Domains control Substrate Recognition and Proteolysis

**DOI:** 10.1038/s41598-019-47494-w

**Published:** 2019-07-29

**Authors:** Salvatore Santamaria, Kazuhiro Yamamoto, Adrienn Teraz-Orosz, Christopher Koch, Suneel S. Apte, Rens de Groot, David A. Lane, Josefin Ahnström

**Affiliations:** 1 From the Centre for Haematology, Imperial College London, Du Cane Road, W12 0NN London, UK; 2 Institute of Ageing and Chronic Disease, William Henry Duncan Building, University of Liverpool, Liverpool, UK; 3 Department of Biomedical Engineering, Lerner Research Institute, Cleveland Clinic, Cleveland, OH USA; 4 Department of Chemistry, Cleveland State University, Cleveland, OH USA

**Keywords:** Proteases, Proteases

## Abstract

ADAMTS (A Disintegrin-like and Metalloproteinase domain with Thrombospondin type 1 Motif)-1, -4 and -5 share the abilities to cleave large aggregating proteoglycans including versican and aggrecan. These activities are highly relevant to cardiovascular disease and osteoarthritis and during development. Here, using purified recombinant ADAMTS-1, -4 and -5, we quantify, compare, and define the molecular basis of their versicanase activity. A novel sandwich-ELISA detecting the major versican cleavage fragment was used to determine, for the first time, kinetic constants for versican proteolysis. ADAMTS-5 (*k*_cat_/*K*_m_ 35 × 10^5^ M^−1^ s^−1^) is a more potent (~18-fold) versicanase than ADAMTS-4 (*k*_cat_/*K*_m_ 1.86 × 10^5^ M^−1^ sec^−1^), whereas ADAMTS-1 versicanase activity is comparatively low. Deletion of the spacer domain reduced versicanase activity of ADAMTS-5 19-fold and that of ADAMTS-4 167-fold. Co-deletion of the ADAMTS-5 cysteine-rich domain further reduced versicanase activity to a total 153-fold reduction. Substitution of two hypervariable loops in the spacer domain of ADAMTS-5 (residues 739–744 and 837–844) and ADAMTS-4 (residues 717–724 and 788–795) with those of ADAMTS-13, which does not cleave proteoglycans, caused spacer-dependent reductions in versicanase activities. Our results demonstrate that these loops contain exosites critical for interaction with and processing of versican. The hypervariable loops of ADAMTS-5 are shown to be important also for its aggrecanase activity. Together with previous work on ADAMTS-13 our results suggest that the spacer domain hypervariable loops may exercise significant control of ADAMTS proteolytic activity as a general principle. Identification of specific exosites also provides targets for selective inhibitors.

## Introduction

**Figure 1:**
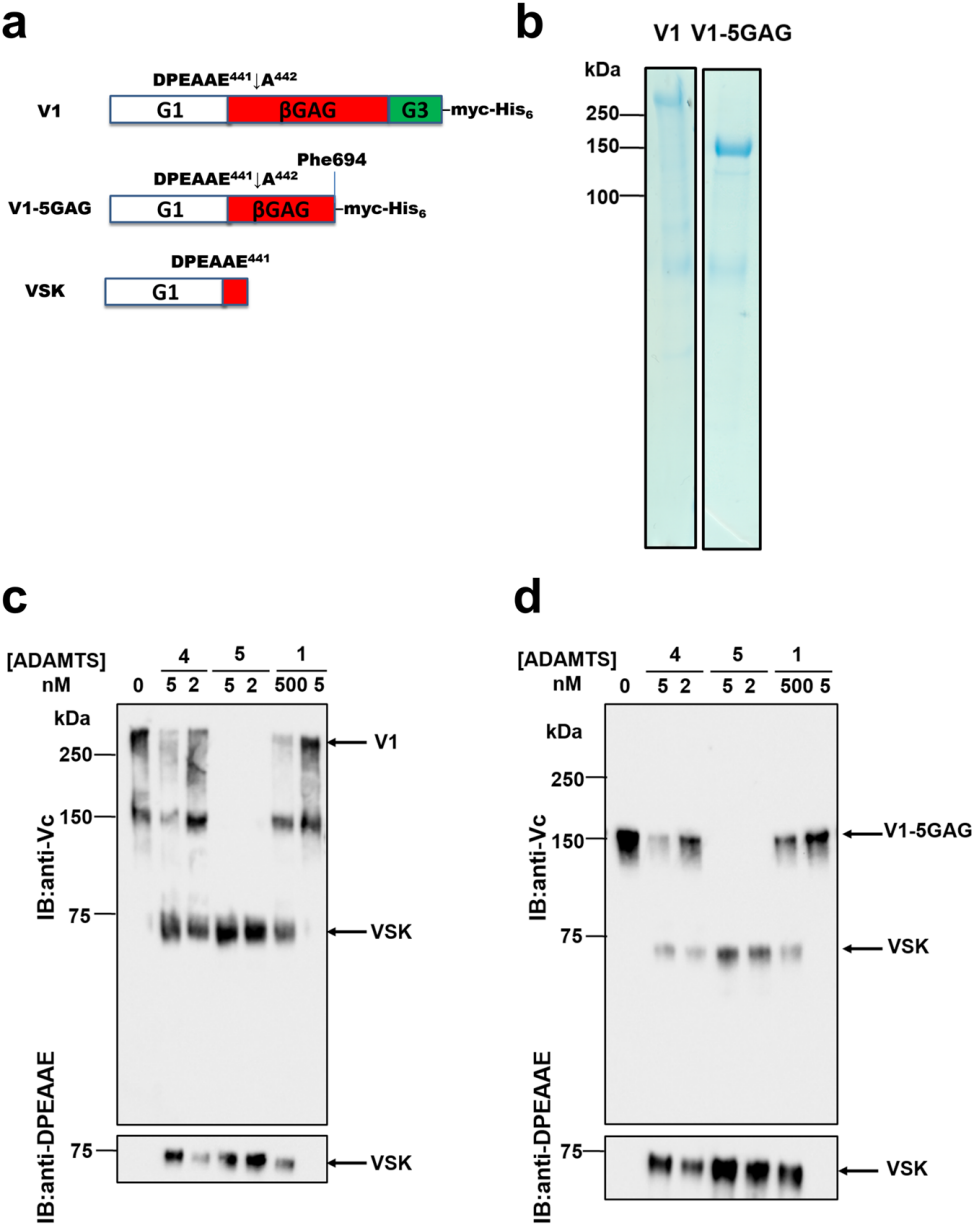
Comparison of the versicanase activity of ADAMTS-1, -4 and -5. (**a**) Domain structures of V1, V1-5GAG and versikine (VSK), the N-terminal cleavage product resulting from ADAMTS-mediated cleavage at Glu441↓442Ala. (**b**) Coomassie staining of purified V1 and V1-5GAG following chondroitinase ABC digestion. Full-length gels are shown in Supplementary Fig. 1. (**c**,**d**) Versicanase activity of ADAMTS-1, -4 and -5. V1 (**c**) and V1-5GAG (**d**) (each 100 nM) were incubated with full length ADAMTS-1, -4 and -5 for 2 h at 37 °C. Samples were deglycosylated, subjected to SDS-PAGE and blotted either with polyclonal anti-Vc or anti-DPEAAE neoepitope antibodies. Enzyme concentrations were chosen with consideration for the relative difference in versicanase activity. IB: immunoblot. Full-length anti-DPEAAE blots are presented in Supplementary Fig. 1.

The ADAMTS (A Disintegrin and Metalloproteinase with Thrombospondin Motif) family comprises 19 secreted zinc endopeptidases in humans. The family members share a common domain structure which comprises a large prodomain, responsible for maintaining the enzyme in an inactive form, a metalloproteinase catalytic domain (Mp), a disintegrin domain (Dis), a thrombospondin-1-like motif (Ts), a cysteine-rich domain (CysR), a spacer domain (Sp) and a variable number of additional C-terminal ancillary domains. A subgroup of ADAMTS proteases, comprising ADAMTS-1, -4, -5, -8, -9, -15, -20, are collectively referred to as proteoglycanases due to their ability to cleave large aggregating proteoglycans (such as versican, aggrecan, brevican and neurocan)^1^. These proteoglycans have a similar structure, comprising a globular N-terminal domain (G1), involved in binding to hyaluronan, a central glycosaminoglycan (GAG) domain containing attachment sites for GAGs, and a globular C-terminal domain (G3). They generate high osmotic pressure due to the negative charges on their GAGs and therefore are essential for the biomechanical properties of the extracellular matrix and tissue swelling. As a consequence, their cleavage and turnover by ADAMTS proteoglycanases has dramatic regulatory consequences for both cells and tissues. For example, genetic deletion of ADAMTS-5 in mice significantly reduced cleavage of aggrecan, the major proteoglycan in articular cartilage, and elicited protection from post-surgical osteoarthritis (OA) and inflammatory-induced arthritis^2,3^. These findings stimulated the development of ADAMTS-5 inhibitors for treatment of OA^4,5^. Another important substrate is versican, which is broadly expressed and is essential for normal embryogenesis^6^, via a role in numerous cellular processes^7^. Four versican isoforms (V0-V3) arise from alternative splicing of two large exons encoding chondroitin-sulfate (Cs) attachment sites, termed αGAG and βGAG^7^. ADAMTS-catalysed proteolysis of the versican V1 isoform, a major component of non-neural tissues, occurs at the Glu441↓442Ala bond within the βGAG region (Fig. 1a), generating an N-terminal fragment named versikine^8,9^. This versicanase activity is associated with ovulation and parturition and major embryological processes such as craniofacial and cardiac morphogenesis, neural crest cell migration and limb development^10^. Moreover, ADAMTS-1, -4 and -5 are implicated in the pathogenesis of cardiovascular diseases^11^. ADAMTS-5 regulates lipoprotein retention in murine models of atherosclerosis via versican turnover^12^. Genetic deletion of ADAMTS-4 in apolipoprotein E (apoE) knock out mice reduced plaque vulnerability and plaque burden^13^, and versican proteolysis by ADAMTS-1 is associated with smooth muscle cell migration^14^. Despite the importance of these findings, the relative activities of these proteases and their structural basis in proteoglycan recognition and proteolysis remain poorly understood.

The ADAMTS-5 Sp domain is known to be strictly required for its aggrecanase activity^15^. Notably, ADAMTS-13, a distantly related protease that cleaves the haemostatic protein von Willebrand Factor (VWF), also requires the Sp for substrate binding^16^. However, specific exosites involved in substrate recognition and proteolysis have not been identified for ADAMTS-5, nor for any other proteoglycanase. So far studies of proteoglycanase activity have been limited to semi-quantitative western blots. Furthermore, low expression levels of the recombinant proteases have inhibited detailed investigations of their enzymatic and binding properties^15,17^.

Here, we use versican as a prototype proteoglycan substrate to define the molecular basis of proteoglycanase activity of ADAMTS-1, -4 and -5. Its recombinant expression^9^ and availability of a well-characterized neoepitope antibody, anti-DPEAAE, against a major ADAMTS cleavage site^8^ provide an opportunity to study proteoglycanase activity in a quantitative manner. Using a newly developed versicanase activity assay and insights gleaned from prior work on ADAMTS-13, we directed this analysis to highly variable loops in their Sp domains. We demonstrate here that these loops contain exosites that are important for proteoglycan recognition and proteolysis.

## Results

### Versicanase activity of ADAMTS-1, -4 and -5

Purified recombinant versican V1 and its variant truncated after Phe694 in the βGAG region^9^, V1-5GAG, provided homogenous preparations of proteoglycan for quantitative measurements (Fig. 1b). Importantly, V1-5GAG contains the Glu441↓442Ala cleavage site (Fig. 1a).

To qualitatively compare the versicanase activities of ADAMTS-1, -4 and -5, we used initially SDS-PAGE followed by western blotting. Purified V1 and V1-5GAG were incubated with purified full-length ADAMTS-1, -4 and -5. The reactions were stopped with EDTA and the digestion products were deglycosylated and subjected to SDS-PAGE and western blotting using either an antibody against the sequence 432-VPKDPEAAEARRG-445 (anti-Vc), which spans the Glu441↓442Ala cleavage site and recognizes both full-length V1/V1-5GAG and versikine^9^, or anti-DPEAAE, which only detects versikine^8^ (Fig. 1c,d). As previously observed^9^, in V1 the anti-Vc antibody detected a band at ~150-kDa (similar size of V1-5GAG), suggesting proteolysis at alternative cleavage sites within the βGAG region during the expression and purification (Fig. 1c). However, this band was not visible in the Coomassie staining (Fig. 1b). The ~150-kDa cleavage band therefore likely represents a minor proportion of V1 preparations, only detected due to the high affinity of the anti-Vc antibody.

At the lowest concentration tested (2 nM), ADAMTS-5 completely converted both substrates into versikine (the ~75-kDa band). In contrast, cleavage by ADAMTS-4 was incomplete even at 5 nM. Very weak versicanase activity was observed for ADAMTS-1, even at concentrations as high as 500 nM.

**Figure 2:**
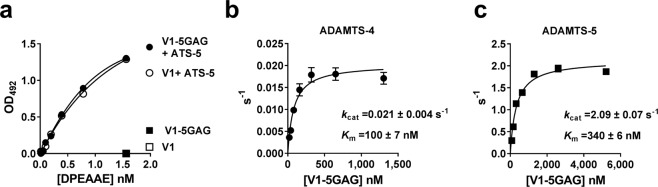
Kinetic constants for the versicanase activity of ADAMTS-4 and -5. (**a**) Representative ELISA standard curves of V1 and V1-5GAG digested with ADAMTS-5 (ATS-5). Following complete digestion of either V1 or V1-5GAG (100 nM) with ADAMTS-5, samples were diluted and incubated on a plate coated with anti-DPEAAE antibodies as reported in the Method Section. (**b**,**c**) Michaelis-Menten curves for proteolysis of V1-5GAG by ADAMTS-4 (**b**) and -5 (**c**). The enzyme (5.5 nM ADAMTS-4, 0.2 nM ADAMTS-5) was incubated with increasing concentrations of substrate. At the indicated time points, an aliquot was taken, proteolysis stopped with EDTA and cleavage products measured by ELISA. Data are plotted as turnover number *versus* substrate concentration and are presented as mean ± SEM (n = 3–4).

For precise quantitation and direct comparison of versicanase activities, we developed a sandwich-ELISA based on the specific recognition of versikine. V1 and V1-5GAG were digested by versicanases essentially as described above. The digested samples were analysed with an ELISA in which the N-terminal versikine fragments were captured by anti-DPEAAE neoepitope antibodies and detected with anti-G1 antibodies. As shown in Fig. 2a the ELISA specifically detected versikine, but not undigested V1/V1-5GAG. Following complete digestion with ADAMTS-5, the two substrates generated very similar binding curves. These results show that the assay could be used to determine the kinetic efficiency of proteolysis of both V1 and V1-5GAG by ADAMTS-1, -4 and -5. This was initially determined by measuring cleavage over time at single substrate concentrations. Both ADAMTS-4 and -5 showed very similar specificity constants (*k*_cat_/*K*_m_) against V1 and V1-5GAG (Table 1), suggesting that the latter substrate contains all binding sites necessary for cleavage at Glu441↓442Ala. Full-length ADAMTS-5 was approximately 18-fold more potent than full-length ADAMTS-4 against both substrates (*k*_cat_/*K*_m _~ 35 × 10^5^ versus 1.86 × 10^5^ M^−1^ s^−1^, Table 1). Due to the very low versicanase activity of ADAMTS-1 (Fig. 1c,d), its specificity constants were not determined. As both V1 substrates were cleaved with similar efficiencies, we used V1-5GAG to determine the turnover numbers (*k*_cat_) and Michaelis-Menten constants (*K*_m_) for ADAMTS-4 and -5 by measuring the initial velocity of proteolysis at different concentrations of substrate (Fig. 2b,c). Comparable *k*_cat_/*K*_m_ values were derived (see also Table 1). ADAMTS-5 achieved its higher versicanase activity by a ~100-fold larger *k*_cat_ which more than compensated for a ~3.5-fold larger *K*_m_ (Fig. 2b,c).

**Table 1:** Kinetic parameters for proteolysis of V1 and V1 5-GAG by ADAMTS -1, -4 and -5 and their deletion variants.

Variant	Substrate	*k*_cat_/*K*_m_^a^ 10^5^ M^−1^ s^−1^	Fold reduction
ADAMTS-4			
WT	V1	1.86 ± 0.37	—
WT	V1-5GAG	2.10 ± 0.28	—
MDTC	V1-5GAG	0.012 ± 0.005**	167
ADAMTS-5			
WT	V1	34.6 ± 5.19	—
WT	V1-5GAG	35.6 ± 3.80	—
MDTCS	V1-5GAG	29.1 ± 3.36	1.2
MDTCS + 2B9	V1-5GAG	7.58 ± 1.50*	4.7
MDTC	V1-5GAG	1.86 ± 0.32*	19
MDT	V1-5GAG	0.23 ± 0.06*^,†^	153
MD	V1-5GAG	0.13 ± 0.03*	276

^a^Values determined by time course experiments at 50 nM substrate concentration.

Results given in nanomolar and expressed as mean ± SEM. *p < 0.05, **p < 0.01, compared to wild-type (WT) enzyme. ^†^p < 0.05 compared with MDTC (n = 3–9).

### Structural determinants of ADAMTS-4 and -5 versicanase activity

**Figure 3:**
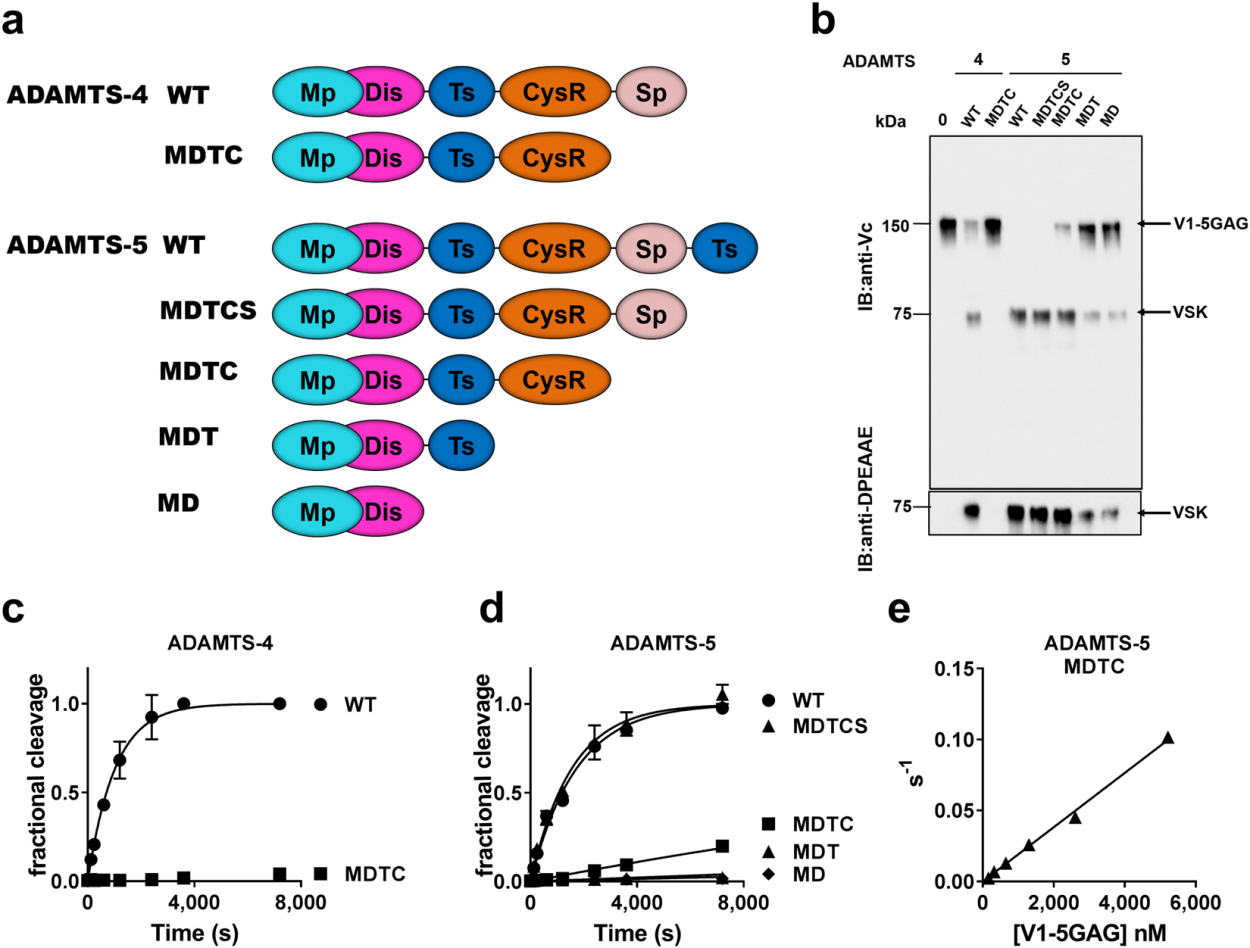
Cleavage of V1-5GAG by ADAMTS-4 and -5 deletion forms. (**a**) A schematic of the different ADAMTS-4 and -5 deletion variants used. (**b**) V1-5GAG (100 nM) was incubated with different variants of ADAMTS-5 and -4 (5 nM) for 2 h at 37 °C. Samples were then deglycosylated, subjected to SDS-PAGE and blotted either with the anti-Vc or anti-DPEAAE antibodies. Full-length anti-DPEAAE blot is presented in Supplementary Fig. 2.VSK, versikine. IB: immunoblot. (**c**,**d**) Time course experiments for cleavage of 50 nM V1-5GAG by ADAMTS-4 (5.5 nM; C) and -5 (0.2 nM; D) variants. The solid lines represent a nonlinear regression fit of the data as described in the Experimental procedures. (**e**) Michaelis-Menten curves for proteolysis of V1-5GAG by ADAMTS-5 MDTC. The enzyme (13 nM) was incubated with increasing concentrations of substrate. At the indicated time points, an aliquot was taken, stopped with EDTA and cleavage products measured by ELISA. Data are plotted as turnover number *versus* substrate concentration and are presented as mean ± SEM (n = 3–6).

The contribution of the ancillary domains of ADAMTS-4 and -5 was explored using previously described domain-deletion variants^15,17,18^ (Fig. 3a). Initially, we used SDS-PAGE followed by western blotting to visualize the versicanase activity of each truncated variant, following a 2 h digestion of V1-5GAG (100 nM) (Fig. 3b). This endpoint experiment indicated that removal of the Sp domain (MDTC variant) had a dramatic inhibitory effect on ADAMTS-4 versicanase activity, as shown by the lack of the anti-DPEAAE reactive cleavage band. For ADAMTS-5, removal of the C-terminal TS-1 domain (MDTCS) had no effect on versicanase activity, whereas removal of the Sp domain (MDTC) resulted in reduced V1-5GAG proteolysis. Further removal of the CysR domain (MDT) severely affected versicanase activity. We then accurately determined the specificity constants of these domain deletion variants in time course experiments. Note that Fig. 3c,d show a comparison of versicanase activities using the same enzyme concentration for all the variants, whereas the values reported in Table 1 were measured using higher concentrations of the variants to induce sufficient cleavage of the substrate, as reported in the Experimental procedures. Deletion of the Sp domain decreased ADAMTS-4 versicanase activity by 167-fold (Fig. 3c and Table 1). Deletion of the C-terminal TS-1 domain of ADAMTS-5 did not alter versicanase activity, whereas deletion of the Sp domain decreased activity by 19-fold **(**Fig. 3d and Table 1). The magnitude of this decrease was underestimated in the western blot shown in Fig. 3b because of the higher substrate and enzyme concentrations used in these experiments as well as the later endpoint (2 h) chosen to stop the reaction (see Experimental procedures). A previously described inhibitory antibody directed against the ADAMTS-5 Sp domain, 2B9^18^, also decreased the versicanase activity of ADAMTS-5 by ~5-fold (Table 1). Since ADAMTS-5 MDTC still retained versicanase activity, the initial velocities at increasing V1-5GAG concentrations were measured to evaluate the consequence of removal of the Sp domain on the kinetic constants. An increase (>30-fold) in *K*_m_ was found, reflecting a decrease in affinity for the substrate (Fig. 3e). Accordingly, the Sp domain mediates the affinity between ADAMTS-5 and proteoglycans. Further deletion of the CysR (MDT) and central TS-1 (MD) domains decreased ADAMTS-5 versicanase activities by 153- and 276-fold, respectively, demonstrating that the other ancillary domains, C-terminal to the Mp/Dis domains, are essential for versicanase activity.

### Identification of hypervariable loops in the ADAMTS-4 and -5 Sp domains bearing essential exosites for versicanase activity

**Figure 4:**
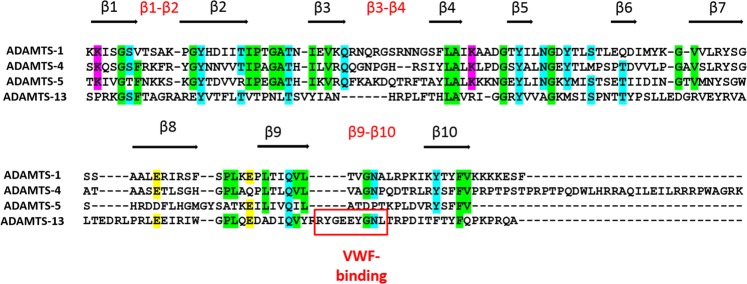
Sequence alignment of the Sp domain of human ADAMTS-1, -4, -5 and -13. Uniprot accession numbers were Q9UHI8 (ADAMTS-1, aa 725–749), O75173 (ADAMTS-4, aa|686–837), Q9UNA0 (ADAMTS-5, aa 732–874) and Q76LX8 (ADAMTS-13, aa 556–685). Percentage identities were 23.9, 17.6 and 14.2%, respectively, compared with ADAMTS-13. Beta strands and β1-β2, β3-β4 and β9-β10 loops are indicated. In ADAMTS-13, the vWF-binding exosite in loop β9-β10 is highlighted. Boxes indicate amino acids conserved in at least three of the four enzymes and are coloured according physicochemical properties (purple, positively charged; yellow, negatively charged; green, apolar; cyan, polar).

The overall fold of the ADAMTS Sp domain was revealed when the structure of the ADAMTS-13 Sp domain was resolved^19^. This showed that the domain consists of ten β-strands in a jelly-roll topology. Of the nine connecting loops, three (β1-β2, β9-β10 and β3-β4) compose a single interface, which in ADAMTS-13 includes a functional exosite (in the β9-β10 loop) responsible for binding to its physiological substrate, VWF^19,20^ (Fig. 4). Interestingly, loops β1-β2, β9-β10 and β3-β4 are highly variable among the otherwise homologous ADAMTS-4 and ADAMTS-5, as well as ADAMTS-1 and the more distantly-related ADAMTS-13 (Fig. 4), suggesting they could potentially be responsible for substrate-specific interactions.

**Figure 5:**
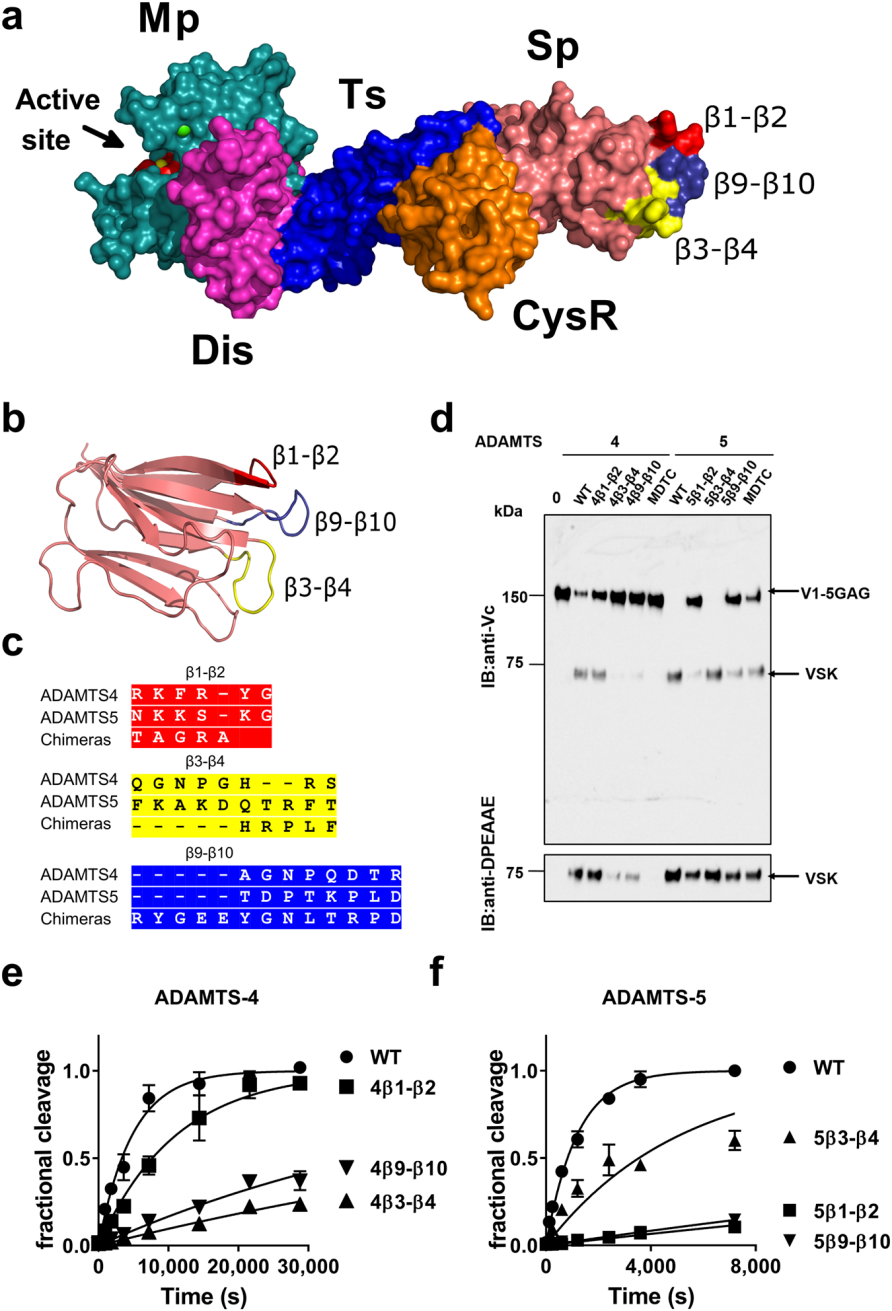
Versicanase activity of ADAMTS-4/13 and -5/13 Sp domain loop chimeras. (**a**,**b**) Molecular model of the ADAMTS-4 Sp domain highlighting loops β1-β2, β3-β4 and β9-β10. Structures of the ADAMTS-4 MD and ADAMTS-13 TCS variants were used as templates. In (**a**) the spatial localisation of the loops relatively to the rest of the molecule is shown, whereas in (**b**) the loops are highlighted in a cartoon model of the isolated Sp domain. (**c**) Sequences of the β1-β2, β3-β4 and β9-β10 loops in wild-type (WT) ADAMTS-4, -5 and the chimeras. (**d**) V1-5GAG (100 nM) was incubated with different variants of ADAMTS-4 and -5 (5 nM) for 2 h at 37 °C. Samples were then deglycosylated, subjected to SDS-PAGE and blotted either with either anti-Vc or anti-DPEAAE antibodies. Full-length anti-DPEAAE blot is presented in Supplementary Fig. 3. VSK, versikine. IB: immunoblot. (**e**,**f**) Time course experiments for cleavage of 50 nM V1-5GAG by ADAMTS-4 (1 nM; **e**) and -5 (0.2 nM; **f**) Sp domain loop chimeras. Data are presented as mean ± SEM (n = 3–6). The solid lines represent a nonlinear regression fit of the data as described in the Experimental procedures.

To get insight into any exosites present in the Sp domains of ADAMTS-4 and ADAMTS-5, we modelled their structures onto that of ADAMTS-13 (Fig. 5a,b): the derived model of ADAMTS-4 is depicted. We hypothesized that loops β1-β2, β9-β10 and/or β3-β4 of ADAMTS-4 and -5 may contain exosites that are important for optimal versicanase activity. To investigate this, we substituted individually these loops in ADAMTS-4 and -5, as identified in the model, with those of ADAMTS-13 (which is unable to cleave proteoglycans)^21^ (Fig. 5c). This substitution approach was considered less likely to cause major conformational changes to the Sp domain than direct deletion of the loop segments. Purified ADAMTS-4/13 and -5/13 chimeras retained the catalytic activities of their wild-type enzymes against small quenched-fluorescent substrates. Like wild-type ADAMTS-4 and -5, their concentrations could therefore be determined by active-site titration with TIMP-3 (see Experimental procedures). These results suggest that mutations in the Sp domain, similarly to the deletion of the whole domain^17^, do not affect catalytic activity against peptide substrates. This is also in agreement with the inhibitory profile observed by anti-Sp antibody 2B9, which specifically inhibits proteoglycanase activity, but not peptidolytic activity^18^. Therefore, using active site-determined concentrations of purified ADAMTS-4/13 and -5/13 chimeras, their abilities to cleave V1-5GAG were compared with their corresponding wild-type and MDTC enzymes using western blot (Fig. 5d). The chimeras ADAMTS-4 β3-β4 and β9-β10 and ADAMTS-5 β1-β2 and β9-β10 showed decreased versicanase activity, comparable with that of their MDTC variants. Next, we performed time course experiments using V1-5GAG, quantified the amount of versikine generated using sandwich-ELISA and determined their specificity constants (Fig. 5e,f and Table 2). In the case of ADAMTS-4, substituting the β3-β4 and β9-β10 loops decreased the versicanase activity by 21- and 13-fold, respectively, whereas substituting the β1-β2 loop had a very modest effect (2.2-fold). For ADAMTS-5, substituting the β1-β2 and β9-β10 reduced the versicanase activity by 43- and 33-fold, respectively, which is within the same magnitude of reduction as the deletion of the Sp domain. Substituting the β3-β4 loop only had a modest effect (3.3-fold) upon activity. From our 3D model (Fig. 5a,b), it is evident that loops β1-β2 and β9-β10 as well as β9-β10 and β3-β4 are contiguous, suggesting that two different, but overlapping, surfaces are involved in the interaction of ADAMTS-4 and -5 with versican.

**Table 2:** Kinetic parameters for proteolysis of V1- 5GAG by ADAMTS -4 and -5 Sp loop chimeras.

Variant	*k*_cat_/*K*_m_^a^ 10^5^ M^−1^ s^−1^	Fold reduction
**ADAMTS-4**		
WT	2.10 ± 0.28	—
β1-β2	0.95 ± 0.21*	2.2
β3-β4	0.10 ± 0.01**	21
β9-β10	0.18 ± 0.03**	12
**ADAMTS-5**		
WT	36.0 ± 3.8	—
β1-β2	0.84 ± 0.05**	43
β3-β4	10.6 ± 1.1**	3.3
β9-β10	1.08 ± 0.06**	33

^a^Values determined by time course experiments at 50 nM substrate concentration. Results given in nanomolar and expressed as mean ± SEM. *p < 0.05, **p < 0.01, compared to wild-type (WT) enzyme (n = 3).

### Shared exosites involved in ADAMTS-5 versicanase and aggrecanase activity

**Figure 6:**
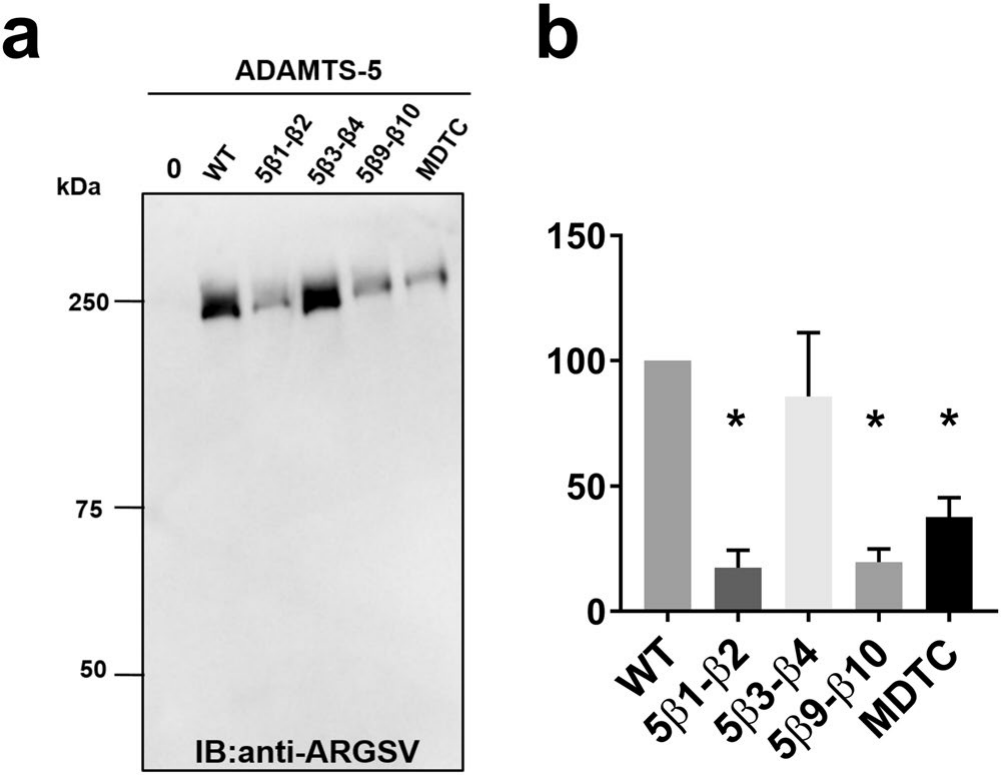
Aggrecanase activity of ADAMTS-5 Sp domain loop chimeras. (**a**) Bovine aggrecan (270 nM) was incubated with ADAMTS-5 (1 nM, 2 h) and their Sp loop variants. Samples were deglycosylated, subjected to SDS-PAGE and detected using anti-ARGSV neoepitope antibody, which specifically detects cleavage at Glu392↓393Ala. (**b**) Densitometric analysis of aggrecan cleavage (n = 3). The anti-ARGSV-reactive bands were quantified and the band in the presence of wild-type (WT) enzyme was set as 100%. The data are presented as average ± SEM; n = 3. Statistical analysis was performed using the unpaired Student’s t-test. p < 0.05 was considered significant. IB: immunoblot.

ADAMTS-5 has emerged as the major aggrecanase in mouse models of OA^2,3^, with its aggrecanase activity being 30-fold higher than that of ADAMTS-4^17^. To investigate whether the ADAMTS-5 exosites involved versican recognition are also important in its aggrecanase activity, we investigated the ability of the chimeric ADAMTS-5/13 loop variants to cleave aggrecan at Glu392↓393Ala, a cleavage site shown to be most detrimental to cartilage integrity^22^ (Fig. 6). As previously shown^15^, removal of the Sp domain (in MDTC) severely reduced aggrecanase activity. Similarly to ADAMTS-5 MDTC, ADAMTS-5/13 β1-β2 and β9-β10 chimeric variants showed decreased aggrecan cleavage at Glu392↓393Ala compared to wild-type ADAMTS-5 (Fig. 6). These results suggest that the same exosites are involved in the recognition of both aggrecan and versican.

## Discussion

Proteoglycanase activity has been implicated in a variety of embryological and pathological processes^2,3,10,11^. The identification of ADAMTS-5 as the major aggrecanase in OA^2,3^ promoted the development of proteoglycanase inhibitors to be used as disease-modifying agents. However, due to lack of knowledge of exosites involved in specific proteoglycanase/proteoglycan interactions, current inhibitors mainly target the well-conserved catalytic domain, resulting in poor selectivity^4^. To date, the majority of selective ADAMTS inhibitors have been limited to monoclonal antibodies^5^. An understanding of the structural requirements for proteoglycanase activity will make these enzymes amenable to selective inhibition through the development of small molecule inhibitors, designed to specifically target exosites.

Versican proteolysis by the ADAMTS family has been shown to play a central role in a variety of embryological processes and in the growth and compaction of the trabeculae in the ventricular myocardium^23,24^. We have used versican, and the development of a quantitative versicanase assay, to study the molecular mechanisms involved in ADAMTS-1, -4 and -5 substrate recognition and proteolysis. These three closely related proteoglycanases have all been identified as versicanases and implicated in the pathogenesis of vascular diseases^11^. Quantification of the intrinsic versicanase activity of ADAMTS-1, -4 and -5 could help elucidate their individual contribution to versican proteolysis. We have, for the first time, determined specificity constants for versicanase activities of ADAMTS-4 and -5 which were in the order of 10^4^–10^5^ M^−1^ s^−1^ (Fig. 2b,c and Table 1) and comparable with those for cleavage of VWF by ADAMTS-13^25–27^. In contrast, the activity of ADAMTS-1 against versican was found to be low compared to ADAMTS-4 and -5, which is in agreement, and explains, previous observations done *in vivo*^8,12^. Out of the three ADAMTS family members investigated, we identify ADAMTS-5 as the most efficient versicanase with a ~18-fold higher proteolytic activity than ADAMTS-4. This was explained by its ~100-fold higher turnover rate. Using semi-quantitative western blots, ADAMTS-5 has also been reported as a 20–30 fold more potent aggrecanase than ADAMTS-4^17^. It appears that out of the three proteoglycanases investigated here, ADAMTS-5 is consistently the most potent *in vitro*. Our findings provide a mechanistic rationale behind some phenotypes observed in genetic models^28^. Deletion of the ADAMTS-5 gene led to versican accumulation and decreased levels of versikine during myofibroblast differentiation^29^ and cardiac valve development^30^. Furthermore, lack of ADAMTS-5 expression lead to increased dilation of the ascending aorta in a model of thoracic aortic aneurysm^31^, and decreased proteoglycan-mediated lipoprotein retention in apoE knockout mice^12^.The consensus from research performed in various *in vivo* models is that expression of multiple ADAMTS proteoglycanases is highly coordinated in situations such as web regression and palatogenesis, where they act cooperatively^10^. The different efficiencies of these proteases may therefore be of relevance by bringing them together to achieve a required threshold of proteolysis required in each specific setting. They are also known to have other substrates^28^, such that some idiosyncrasy is necessary, but preservation of shared characteristics may ensure the necessary proteoglycanase activity. The low versicanase activity observed for ADAMTS-1, which is nonetheless significantly associated with versican proteolysis *in vivo*^24,32^, may result from the absence of a cofactor, such as fibulin-1^23,33^ in the current study.

The versicanase assay allowed us to dissect the contribution of each domain for versicanase proteolysis using a series of domain-deletion variants of ADAMTS-4 and -5^15,17,18^ (Fig. 3 and Table 1). Since ADAMTS-5 MD is inactive against versican^10^ and the crystal structure of ADAMTS-5 MD indicates that the Mp and Dis domains are structurally integrated^34^, we focused on the ancillary TCS(T) domains of ADAMTS-4 and -5. We found that deletion of the Sp domain reduced the versicanase activity of ADAMTS-4 by ~170-fold, whereas for ADAMTS-5 both the Sp and CysR domains needed to be deleted to observe a similar reduction. Complementary studies of aggrecanase activity using semi-quantitative western blots showed that deletion of the Sp domain in ADAMTS-4 and -5 reduced activities by 20-fold^17^ and 4-fold^15^, respectively. Further deletion of the ADAMTS-5 CysR domain reduced its aggrecanase activity by ~200-fold^15^, suggesting similarities in substrate recognition between aggrecan and versican. Our previous research has suggested that the ADAMTS-5 Sp interacts with the aggrecan protein core^18^, whereas its CysR has been shown to be important in contacting GAGs in aggrecan^15^. In ADAMTS-13, the CysR and Sp domains bind to adjacent sequences on VWF^26^. Similarly to VWF recognition of ADAMTS-13, dual domain recognition of substrate occurs also for the ADAMTS-5 interaction with versican and aggrecan.

Since the Sp is essential for the versicanase activity of ADAMTS-4 and plays an important role in that of ADAMTS-5, we focused on this domain in our effort to identify exosites. We substituted loop segments (loops β1-β2, β9-β10 and β3-β4) of the Sp domain for those in ADAMTS-13, which does not cleave proteoglycans^21^ (Fig. 5 and Table 2). The rationale behind this approach was that ADAMTS-13 loop β9-β10 includes a functional exosite responsible for binding to its physiological substrate, VWF^19,20^. Furthermore, the β1-β2 loop in ADAMTS-4 and -5 contains a GAG binding site which could be involved in versican interaction^35,36^. Sp domain loops β3-β4 (aa 717–724) and β9-β10 (aa788–795) in ADAMTS-4 and loops β1-β2 (aa 739–744) and β9-β10 (837–844) in ADAMTS-5 emerged as important exosites. Intriguingly, we showed that for ADAMTS-5 these exosites are important also for aggrecan cleavage at Glu392↓393Ala (Fig. 6), a cleavage site that it is most detrimental for cartilage integrity^22^. The presence of hypervariable sequences in the Sp loops of different ADAMTS family members (Fig. 4) suggest that these sequences may be responsible for substrate-specific interactions. Furthermore, the identification of exosites involved in substrate binding suggests potential targets for future development of ADAMTS-4 and -5 inhibitors.

## Methods

### Expression plasmids and site-directed mutagenesis

Constructs coding for human ADAMTS-4 and -5 (both full-length and their carboxyl-terminal deletion mutants, Fig. 3a) with a C-terminal FLAG (DYKDDDDK) tag in pCEP4 vector were described previously^15^. ADAMTS-1 was cloned into the pCEP4 vector in-frame with a C-terminal FLAG tag using PCR. The PCR was performed using full-length ADAMTS-1 cDNA as a template and amplified by KOD Hot Start DNA Polymerase (Merck) using forward primer, 5′-ACTGGT*ACCACCATG*CAGCGAGCTGTG-3′ (ADAMTS-1 FW) containing a KpnI restriction site (underlined) and a Kozak consensus sequence (italic); reverse primer, 5′- CTGCCTCGAGCTA*TTTATCATCATCATCTTTATAATCAC*TGCATTCTGCCATTGT-3′ (ADAMTS1-REV FLAG) containing a *Xho*I restriction enzyme site (underlined), a stop codon and the FLAG epitope (in Italics). PCR was carried out for 35 cycles of denaturation (60 s at 94 °C), annealing (60 s at 55 °C), and extension (3 min 30 s at 72 °C). The PCR products were digested with *Kpn*I and *Xho*I (New England Biolabs, 2 h, 37 °C) and ligated into pCEP4 vector using T4 DNA Ligase (16 h, 16 °C).

For generation of ADAMTS-4 and -5 mutants, the sequences of ADAMTS-4 and -5 were first cloned into a pEGFP-N1 vector using the primers: ADAMTS-4 forward 5′-ACTGGTACCACCATGTCCCAGACAGGCTCG-3′; ADAMTS-5 forward, 5′-ACTGGTACCACCATGCTGCTCGGGTGGGCG-3′; FLAG reverse, 5′-CTGCGCGGCCGCCTATTTATCATCATCATCTTTATAATC-3′ (*Kpn*I and *Not*I restriction sites are underlined, stop codon is in bold text and the FLAG epitope (in Italics). PCR was carried out as described above. PCR products were digested with *Kpn*I and *Not*I (2 h, 37 °C) and ligated into pEGFP-N1 using T4 DNA Ligase.

ADAMTS-4/13 and -5/13 Spacer (Sp) domain loop chimeras were generated by reverse PCR using ADAMTS-4 and -5 as template and the following set of primers (the inserted ADAMTS-13 sequence is underlined): 4KL1 forward, 5′-AGAGCGTACAACAATGTGGTCACTATC-3′; 4KL1 reverse, 5′-GCCAGCTGTGAAG GAGCCTGACTGCTT-3′;4KL2 forward, 5′-CTCTTCATCTACTTGGCCCTGA-3′;4KL2 reverse, 5′-AGGCCTGTGCTGCCGGACAAGAATGTG-3′;4KL3 forward, 5′-GGCAACCTCAC CCGCCCAGACCTCCGATACAGCTTCTT-3′;4KL3 reverse, 5′-ATACTCCTCGCCATACCGCACTAGGACTTGCAG-3′;5KL1 forward, 5′-AGAGCGTACACTGACGTGGTGAGG-3′;5KL1 reverse, 5′-GCCAGCTGTAAAGGTTCCAACAATCTT-3′;5KL2 forward, 5′-CTCTTCGCCTATTTAGCCCTGAAA-3′;5KL2 reverse, 5′-AGGCCTGTGCTGTCGAACTTTTATGTG-3′;5KL3 forward, 5′-GGCAACCTCACCCGCCCAGACGTCCGTTATAGCTTTTTT-3′;5KL3 reverse, 5′-TACTCCTCG CCATACCGTGCAAGAATCTGCAC-3′. The PCR was carried out as above and the PCR products were treated with T4 kinase and T4 ligase.

All the constructs were sequenced to confirm that no point mutations were introduced during PCR.

A versican V1 plasmid coding for full-length mature human V1 inserted into pSecTagA after the Igκ secretory leader sequence and the C-terminally-truncated variant V1-5GAG, comprising amino acids 21–694 with C-terminal tandem myc/His_6_ tags were described previously^15,37^.

### Cell lines and transfections

Stable cells lines HEK293 EBNA expressing ADAMTS-4 and -5 variants were grown in modified Eagle’s medium (MEM) (Sigma) supplemented with 10% fetal calf serum, 2 mM L-glutamine (Sigma), 1% non-essential amino acids and penicillin/streptomycin (100 U/mL) (Invitrogen). For ADAMTS-1 and ADAMTS-4 and -5 Sp domain loop chimeras, transient transfections were carried out in HEK293T cells (ATCC) using linear polyethylenimine (PEI). Heparin (from porcine mucosa, Sigma, 200 μg/mL) was added 4 h post-transfection.

### Protein expression and purification

ADAMTS-1, -4 and -5 were expressed in serum-free MEM containing 200 μg/mL heparin (Sigma) to extract extracellular matrix-bound enzyme, concentrated using a Lab scale TFF system (Merck) and purified using anti-FLAG affinity resin (Cat. n.:A2220, Sigma) as previously described^17^. Briefly, after loading the medium, the column was washed with 1 M NaCl to remove heparin^38^, and the bound protein was eluted with 200 μg/ml FLAG peptide (Cat. n.:F3290, Sigma). Proteins were separated by SDS-PAGE and analysed by western blot using the following primary antibodies: anti-FLAG M2 mouse monoclonal antibody (Cat. n.:1804, Sigma; 1:1000), anti-ADAMTS-4 rabbit polyclonal antibodies directed against the Pro/MP domains (aa 52–315) (Cat. n.:185722, Abcam; 1:500), anti-ADAMTS-5 rabbit polyclonal antibodies directed against the MP domain (aa 338–368) (Cat. n.:135656, Abcam; 1:500). Purity was assessed by silver-stain. Concentrations of active ADAMTS-4 and -5 and their variants were determined under kinetic equilibrium conditions by active-site titrations with known concentration of TIMP-3 (Bio-Techne, Cat. n.:973-TM-010, Bio-Techne)^39^ using quenched fluorescent peptides (custom synthesized by Bachem) carboxyfluorescein-A-E↓L-N-G-R-P-I-S-I-A-K-*N*,*N*,*N*′,*N*′-tetramethyl-6-carboxyrhodamine [Fam-AE↓LQGRPISIAKTamra, ‘↓’ indicates the cleavage site] (for ADAMTS-4) and o-aminobenzoyl-T-E-S-E∼S-R-G-A-I-Y-(N-3-[2,4-dinitrophenyl]-L-2,3-diamino-propionyl)-K-K-NH_2_ [Abz-TESE↓SRGAIY-Dpa-KK] for ADAMTS-5 as previously reported^40–42^. Final substrate concentrations were 1 μM and 20 μM for ADAMTS-4 and -5, respectively. Due to lack of suitable quenched-fluorescent substrates for ADAMTS-1, total enzyme concentration was measured by optical absorbance at 280 nm using extinction coefficient of 1.373 (E1%, 1 cm) as predicted by the ProtParam Tool (ExPasy).

V1 and V1-5GAG were transiently transfected using PEI in OPTIMEM (Invitrogen) containing 2 mM CaCl_2_. As previously shown^9^, V1-5GAG, but not V1, was detectable by anti-6x His tag antibody on western blot analyses, despite the cloning of the V1-ORF in-frame with the myc-His6 tag (data not shown). This could possibly be due to proteolytic loss of the tag in the latter construct. Therefore, V1 was purified using HiTrap DEAE Sepharose (GE Healthcare). After sample loading, the column was washed with 10 CV 20 mM Tris-HCl pH 7.4, 250 mM NaCl and eluted using a linear gradient of NaCl (250–1000 mM) over 10 column volumes (CV). V1-5GAG was purified using a Ni-sepharose column (GE Healthcare) equilibrated with 3 CV TBS (20 mM Tris-HCl pH 7.4, 150 mM NaCl). Following binding, the column was washed with TBS containing 10 mM imidazole and bound proteins were eluted using a linear gradient (10–300 mM) of imidazole. Eluted fractions containing recombinant proteins were subjected to SDS-PAGE, pooled, concentrated on Amicon Ultra spin columns (100 kDa cut-off) and dialyzed extensively against TBS. Substrates were stored at −80 °C.

### Evaluation of proteoglycanase activity by western blot

Qualitative analysis of the versicanase activity of wild-type ADAMTS-1, -4 and -5 and their domain deletion mutants was carried out by incubating each enzyme (2–5 nM ADAMTS-4 and -5; 5–500 nM ADAMTS-1) with either V1-FL or V1-5GAG (100 nM) in TNC-B buffer (50 mM Tris-HCl pH 7.4, 150 mM NaCl, 5 mM CaCl_2_, 0.02% (w/v) NaN_3_, 0.05% (v/v) Brij® 35) at 37 °C. Sub-samples were removed and reactions were stopped at different time points (0–24 hours) with ethylenediaminetetraacetic acid (EDTA, 25 mM) in deglycosylation buffer (50 mM sodium acetate, 25 mM Tris HCl pH 8.0) containing 0.1 U/mL chondroitinase ABC (AMSbio) for 16 h at 37 °C. Digestions were analysed using western blotting under reducing conditions (5% β-mercaptoethanol) on 4–12% Bis-Tris NuPage Gels (Thermo Fisher). Versican fragments were detected with either anti-Vc rabbit polyclonal antibody against the βGAG region (1 μg/ml)^9^ or neoepitope anti-DPEEAE rabbit polyclonal antibody (Cat n.: PA1-1748A, Life Technologies).

Aggrecan digestion assays were performed as previously described^18^. Briefly, aggrecan from bovine articular cartilage (270 nM) (Cat. n.: A1960 Sigma, numbering according to Uniprot accession number: P13608) was incubated with different concentrations of ADAMTS enzymes as reported in the text in TNC-B buffer at 37 °C for 2 h. The reaction was stopped with EDTA buffer and aggrecan was incubated with 0.1 U/mL of chondroitinase ABC and keratanase (endo-beta galactosidase, Cat. n.: G6920, Sigma) overnight at 37 °C to remove GAG chains. Samples were analysed by western blot under reducing conditions and cleavage products were detected using mouse monoclonal BC-3 antibody which detects aggrecan cleavage at the Glu392↓Ala393 bond (Cat n.: MA316888, Life Technologies). Immobilon Chemiluminescent HRP substrate (Merck Millipore) was detected with a Chemidoc Touch Imaging system (Bio-Rad) and band intensities were measured using Image lab software version 5.2.1 (Bio-Rad).

### Quantification of versicanase activity by sandwich ELISA

For quantitative analysis of versican cleavage products, 96-well Maxisorp plates (Nunc) were coated with 5 μg/mL anti-DPEEAE neoepitope antibody (Cat n. PA1-1748A, Life Technologies) in carbonate buffer pH 9.6 (16 h, 4 °C). Washing steps were performed in triplicate with 300 μL phosphate buffered-saline (PBS) containing 0.1% Tween-20 between each step. Plates were blocked with 3% bovine serum albumin (BSA)/PBS for 2 h, at room temperature (RT). The samples from the digestion experiments were diluted in 3% BSA/PBS and added to the plate (100 μL, 2 h, RT). Bound DPEEAE-containing versican fragments were detected using anti-G1 monoclonal antibody (Cat n. ab171887, Abcam, 3 μg/mL in 0.5% BSA/PBS) (1.5 h, RT), followed by horseradish peroxidase (HRP)-conjugated anti-mouse antibodies (DAKO, 2.4 μg/mL, 1 h, RT). The assay was developed by addition of o-phenylenediamine dihydrochloride (OPD, Cat n. 34006, Sigma) for 10 minutes and reactions were stopped with 2 M H_2_SO_4_. The absorbance was read at 492 nm. For the determination of specificity constants, ADAMTS-5 (final concentration 1 nM) or -4 (5.5 nM) were incubated with different concentrations of V1-5GAG (0–3200 nM) at 37 °C in TNC-B buffer. At different time points (0–14 min), sub-samples were removed and reactions were stopped with EDTA. For each dilution, the amount of neoepitope generated was derived from a standard curve (0–1.56 nM) of V1-5GAG completely digested with ADAMTS-5. Initial velocities were calculated from the concentration of versikine generated as a function of reaction time. These were divided by the enzyme concentration to derive the turnover number in s^−1^. Turnover numbers were then plotted against substrate concentrations and fitted to the Michaelis-Menten equation using GraphPad Prism Software to determine *k*_cat_ and *K*_m_ values.

For independent quantification of the specificity constants (*k*_cat_/*K*_m_), digestion reactions were set up exactly as in the preceding paragraph, except that 50 nM V1-5GAG was used and data were analysed as previously described^26^. Specificity constants reported in Tables 1 and 2 were measured at the following enzyme concentrations: ADAMTS-4 WT: 5.5 nM; 4MDTC: 55 nM; 4β1-β2, 4β3-β4 and 4β9-β10 1 nM; ADAMTS-5 WT and MDTC: 0.2 nM; 5MDTC: 2.7 nM; 5MDT: 23 nM; 5MD: 26 nM; 5β1-β2 and 5β9-β10 2.7 nM; 5β3-β4:0.4 nM. All assays were performed at least 3 times using different batches of recombinant enzymes.

### Statistical analysis

Data are presented as mean ± SEM of at least three independent experiments and were analyzed by GraphPad Prism Software. Statistical analysis was performed using by Mann-Whitney test. p < 0.05 was considered significant.

### Molecular modelling of the ADAMTS-4 and ADAMTS-5 Spacer domains

To predict the secondary and tertiary structure of the ADAMTS-4 and ADAMTS-5 Sp domains they were modelled with the Bioinformatics Toolkit of the Max Planck Institute for Developmental Biology, Tübingen, Germany^43,44^, using the structure of ADAMTS-13 (3GHM) as template. Molecular graphics were produced with an open source version of Pymol precompiled by Christoph Gohlke (University of California, Irvine).

## Supplementary information

Supplementary Information

## Data Availability

Reagents and data presented in this study are available from the corresponding authors upon request.
