# Supplementary material for: Exosites in Hypervariable Loops of ADAMTS Spacer Domains control Substrate Recognition and Proteolysis
Source: Sci Rep. 2019 Jul 29;9:10914. doi: 10.1038/s41598-019-47494-w (PMC6662762; doi:10.1038/s41598-019-47494-w)

SUPPORTING INFORMATION

Exosites in Hypervariable Loops of ADAMTS Spacer Domains control Substrate Recognition and Proteolysis

Salvatore Santamaria[1,*], Kazuhiro Yamamoto[2], Adrienn Teraz-Orosz[1], Christopher Koch[3,4], Suneel S. Apte[3], Rens de Groot[1], David A. Lane[1] and Josefin Ahnström[1, *]

Supplementary Figure 1. Full-length gels of Figure 1B and anti-versikine blots of Figure 1C and 1D. Red squares represent the part of the images showed in Figure 1.

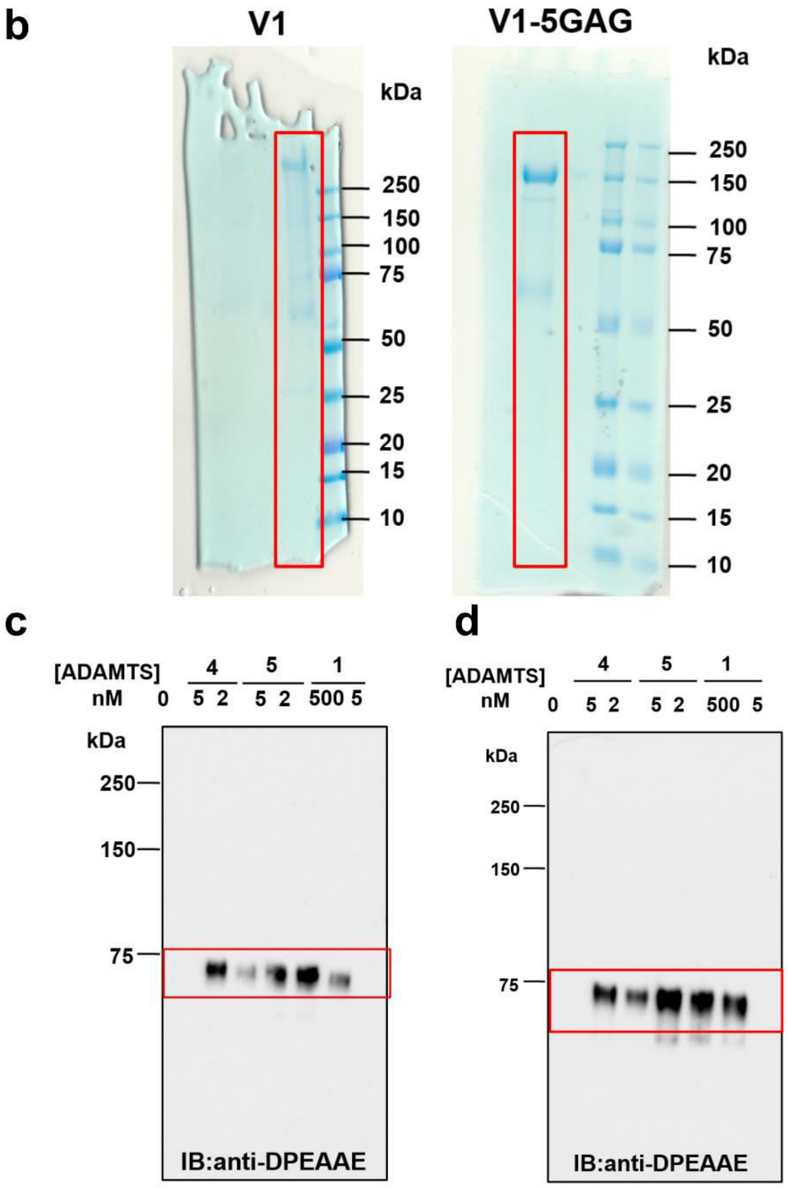

Supplementary Figure 2. Full-length anti-DPEAAE blots of Figure 3B

Red squares represent the part of the image showed in Figure 3B.

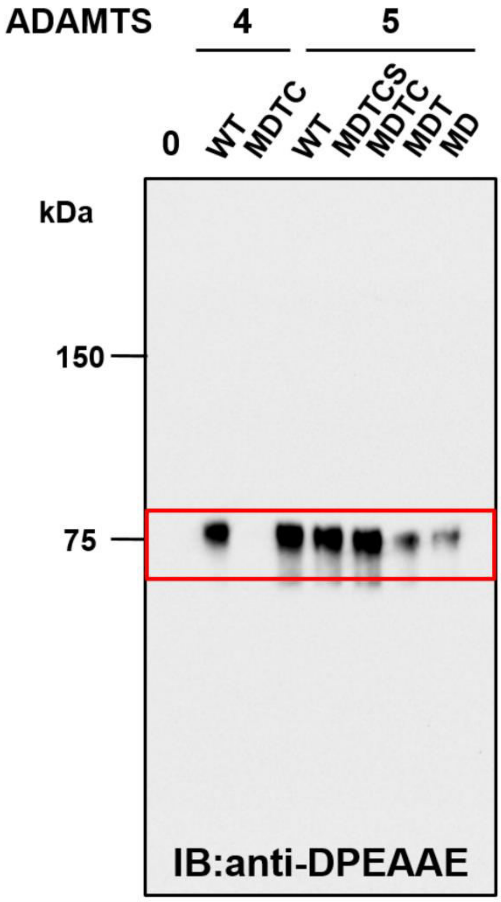

Supplementary Figure 3. Full-length anti-DPEAAE blots of Figure 5D

Red squares represent the part of the image showed in Figure 5D.

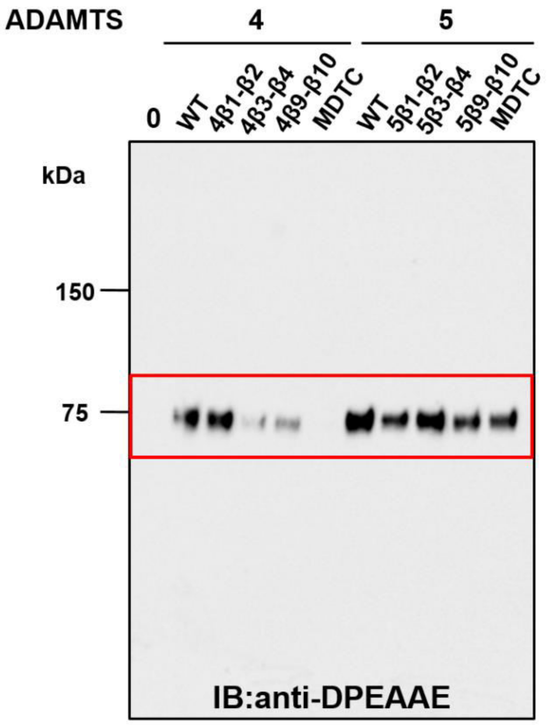

Supplement: Supplementary file 1 — Supplementary Information [file 41598_2019_47494_MOESM1_ESM.pdf]
